# Characterization of the caspase family in zebrafish

**DOI:** 10.1371/journal.pone.0197966

**Published:** 2018-05-23

**Authors:** Olivia Spead, Tine Verreet, Cory J. Donelson, Fabienne E. Poulain

**Affiliations:** Department of Biological Sciences, University of South Carolina, Columbia, South Carolina, United States of America; University Zürich, SWITZERLAND

## Abstract

First discovered for their role in mediating programmed cell death and inflammatory responses, caspases have now emerged as crucial regulators of other cellular and physiological processes including cell proliferation, differentiation, migration, and survival. In the developing nervous system, for instance, the non-apoptotic functions of caspases have been shown to play critical roles in the formation of neuronal circuits by regulating axon outgrowth, guidance and pruning. How caspase activity is spatially and temporally maintained at sub-lethal levels within cells remains however poorly understood, especially in vivo. Thanks to its transparency and accessibility, the zebrafish offers the unique ability to directly visualize caspase activation in vivo. Yet, detailed information about the caspase family in zebrafish is lacking. Here, we report the identification and characterization of 19 different caspase genes in zebrafish, and show that caspases have diverse expression profiles from cleavage to larval stages, suggesting highly specialized and/or redundant functions during embryonic development.

## Introduction

Caspases are highly conserved intracellular cysteine-dependent proteases best known for their critical role in mediating apoptosis and inflammatory responses [1, 2]. As transducers and executioners of programmed cell death, caspases are especially important during development, when the elimination of unnecessary cells contributes to tissue morphogenesis [3]. In the nervous system, for instance, apoptosis has been detected in neural precursor cells, postmitotic neurons and glial cells [4], and a lack of caspase-3 or -9 has been shown to cause brain hyperplasia and neural overgrowth in mice [5–8]. More recently, caspases have also emerged as central mediators of non-apoptotic signaling pathways regulating a large variety of cellular and physiological functions [9–12]. How caspase activation is induced and maintained at sub-lethal levels within cells remains however poorly characterized.

Caspases are synthesized as zymogens that become activated upon oligomerization or cleavage at specific aspartate residues. They comprise an N-terminal prodomain of varying size and a catalytic CASc domain that includes a large P20 and a small P10 subunits. So far, 12 caspases have been identified in human (Caspases-1 to 10, -12, and -14, with -12 being inactive due to deleterious mutations), and additional caspases including Caspases-11 and -13 (murine and bovine orthologs of human Caspase-4, respectively), -15, -16, -17 and -18 have been detected in other vertebrates [13–17]. Based on their functional and structural similarities, caspases have generally been classified as inflammatory (Caspases-1, -4, -5 and -12) and initiators (Caspases-2, -8, -9 and -10) or executioners of apoptosis (Caspases-3, -6 and -7). However, this subdivision has progressively become obsolete with the discovery of functions in additional processes including cell proliferation [18], differentiation [19, 20], motility and migration [11, 21], survival [22] and tissue regeneration and repair [23].

Essential non-apoptotic functions of caspases have especially been demonstrated in the developing and mature nervous system [10, 12, 24, 25]. Caspase-3, for instance, has been implicated in the regulation of neural stem cell differentiation [26], Bergman glia differentiation [27, 28] synaptic plasticity, learning and memory [29–33]. More recently, caspases have also been identified as important regulators of axon outgrowth and pathfinding. The role of caspase activity in axon guidance was first suggested by the observation that growth cone responses to netrin or lysophosphatidic acid were blocked in the presence of caspase inhibitors in vitro [34]. Since then, caspases have been shown to regulate NCAM-dependent axon outgrowth [35], axon targeting in the auditory brainstem and the olfactory bulb [36, 37], and retinal axon arborization [38]. Caspases have also emerged as playing a crucial role in the refinement of neuronal connectivity by regulating axonal and dendritic pruning [39–44]. For instance, pruning of retinal axons projecting to the superior colliculus is delayed in mice lacking caspase-3 or -6 [42, 43].

How the non-apoptotic activity of caspases is spatially and temporally induced and restricted in cells or axons is not yet well understood. Several mechanisms have been proposed, including subcellular regulation by inhibitors such as XIAP [45] or post-translational modifications [12]. Yet, our understanding of when, where and how caspases are locally activated and controlled in vivo remains rudimentary due to a limited number of models suitable for high resolution in vivo imaging. Thanks to their advantageous accessibility and transparency, zebrafish embryos offer the unique ability to directly visualize axon development and degeneration in vivo and address these questions [46, 47]. The recent use of genetically encoded biosensors to detect caspase activation in real time in this model has revealed an important function of Caspases-3 and -9 in axon remodeling [38], and will likely provide new insight into the fine spatio-temporal activation of caspases in other contexts. Yet, detailed information about caspases in zebrafish is surprisingly lacking. To gain insight into the functions of caspases during axon guidance and pruning in vivo, we first carried out a comprehensive analysis of the caspase family in zebrafish. We report here the identification and characterization of 19 different caspase genes including known orthologs of human caspases as well as new members of this family. We also show that zebrafish caspases have distinct expression patterns during development, suggesting both specific and conserved functions among vertebrates.

## Material and methods

### Zebrafish husbandry

This research was approved by the University of South Carolina Institutional Animal Care and Use Committee (IACUC) (protocol number: 2398-101289-111717). Wild type (WT) embryos (Tübingen and AB strains) were obtained from natural matings, raised at 28.5°C in E3 medium (5 mM NaCl, 0.17 mM KCl, 0.33 mM CaCl_2_, and 0.33 mM MgSO_4_) in the presence of 150 mM of 1-phenyl-2-thiourea (PTU) (Sigma) to prevent pigment formation, and staged by age and morphology [48]. Embryos were anaesthetized in tricaine (Western Chemicals) before fixation or RNA extraction.

### Identification and cloning of *caspase* coding sequences

GenBank and the Ensembl *Danio rerio* (GRCz10) databases were used to identify genomic loci for all zebrafish caspase genes. *Mus musculus*, *Homo sapiens*, *Bos taurus*, and *Gallus gallus* caspase gene sequences were blasted against the databases and the zebrafish sequences identified were confirmed for the presence of a CASc domain (SMART accession number SM00115). Zebrafish mRNA was isolated from embryos at cleavage, blastula sphere, gastrula shield, gastrula bud, pharyngula prim-5 (24 hours-post-fertilization (hpf)), long-pec (48 hpf), protruding mouth (72 hpf) and larval day 4 (96 hpf) stages using Trizol and the RNeasy mini kit (Qiagen), and cDNA was prepared from RNA using the SuperScriptIII First-Strand Synthesis system (Invitrogen). Full length primers (Table 1) were used to amplify zebrafish caspase cDNAs. Amplicons were subcloned into PCRII-TOPO (Invitrogen) and sequenced to verify gene identity and confirm sequence orientation for the generation of sense and antisense RNA probes. Protein sequences were analyzed using the Eukaryotic Linear Motif (ELM) resource prediction tool and Motif Scan (MyHits, SIB, Switzerland) to identify and annotate functional domains [49].

**Table 1:** Primers used for caspase cloning.

Gene name	forward primer	reverse primer
*casp1*	ATGGCCAAATCTATCAAGG	TCAGAGTCCGGGGAA
*casp19a*	ATGGAGGATATTACCCAG	TCACAGTCCAGGAAAC
*casp19b*	ATGGAGGATATTACCAAGTTG	TCACTGTCCAGGGAAC
*casp23*	AATCGTCGTTTAGCGCTTTAG	GCAGATATATATTGCACTTGCTATG
*casp2*	ATGTTGGGAGAGTGC	TTAGTTGCTGGGGTAG
*casp9*	ATGGAGCAGAAACACAG	TCATGACTGTGAAGACTG
*casp8a*	ATGGATCCTCAGATCTTTCACG	TCAGTCTATGGGCAGCACT
*casp8b*	ATGGATAAAACTAGTAATCCTA	TTAGGAGACTCCATTCAT
*casp10*	GACATGGACATGTGTTTTCAGAG	GAGCATCATCAAGGAAGCC
*casp20*	ATGAGTAAAAAGGAATCAACTC	TCAGTTATTCACTGGCG
*casp22*	ATGGCAGATCAACTTTTGG	CTGTTTAAGAGAACCGGC
*casp3a*	ATGAACGGAGACTGTGTG	TTAAGGAGTGAAGTACATCTCTTTG
*casp3b*	ATGTCGCACGTGAAACCA	TTATTTAGGGAAGTAGAGTTCTTTGG
*casp6a*	ATGGCAAGTCACACT	TTACTTTTTGGGCCTG
*casp6b*	ATGGCAACTAACACCAGAAGC	CTATTGGATCTGAGTATTGTCTCTG
*casp6c*	ATGCACCACAACAAATCAATAATG	GTAGGGATTAGGTATGGATTAG
*casp7*	ATGAATAAAGAAGCCCTTACTTCC	TCAGTTGAAGTAGAGCTCTTTAG
*casp21*	ATGAGTTTACAAGCTTCTAAAGAC	TTACAATTTCTTATTCTTCTCTGCAAC
*casp17*	CAACACAAGCACTAATGTCAG	TCCTCATTTCTGTGATCTTCAG

### Sequence comparison and phylogeny

Zebrafish caspase protein sequences were compared to the following protein sequences using the MatGAT (Matrix Global Alignment Tool) software [50] with a BLOSUM 62 scoring matrix (gap opening and gap extending penalties of 4): *Homo sapiens* Caspase-1 (NP_150634), Caspase-2 (NP_116764), Caspase-3 (NP_116786), Caspase-4 (NP_001216), Caspase-5 (NP_001129584), Caspase-6 (NP_001217), Caspase-7 (NP_001253986), Caspase-8 (NP_001073594), Caspase-9 (NP_001220), Caspase-10 (NP_116759), Caspase-12 (NP_001177945), and Caspase-14 (NP_036246), *Bos Taurus* Caspase-15 (NP_001029681), *Mus musculus* Caspase-16 (XP_003945628), *Gallus gallus* Caspase-17 (UniProt A9YDV3), and Caspase-18 (NP_001038154). Phylogenetic analyses were conducted using the Mega 7 software [51] and included the following additional sequences: *Gallus gallus* Caspase-1 (XP_003642432.2), Caspase-2 (NP_001161173), Caspase-3 (NP_990056.1), Caspase-6 (NP_990057), Caspase-7 (Uniprot F1NV61), Caspase-8 (NP_989923.1), Caspase-9 (Uniprot F1NL59), Caspase-10 (XP_421936.4), *Latimeria chalumnae* Caspase-1 (Uniprot H3B2V3), Caspase-2 (Uniprot H3A019), Caspase-3 (Uniprot H3ACL5), Caspase-6 (Uniprot H2ZXX5), Caspase-7 (Uniprot M3XIX0), Caspase-8 (Uniprot H3A526), Caspase-9 (Uniprot H3BFW5), Caspase-10 (Uniprot H2ZXE8), Caspase-14l (XP_014344655.1), Caspase-17 (Uniprot H3AXG0), Caspase-18 (H3A2R2), *Oryzias latipes* Caspase-1 (Uniprot H2LPF5), Caspase-2 (XP_011483724.1), Caspase-3a (NP_001098168.1), Caspase-3b (NP_001098140.1), Caspase-6 (Uniprot H2MXM9), Caspase-6l1 (XP_023813211.1), Caspase-6l2 (XP_023813213.1), Caspase-7 (XP_023805391.1), Caspase-8 (NP_001098258.1), Caspase-9 (Uniprot H2LBD7), Caspase-17 (Augustus g31162.t1), Caspase-20 (XP_023820994.1), *Takifugu rubripes* Caspase-1 (Uniprot H2SKU3), Caspase-1l (Uniprot H2UDK1), Caspase-2 (H2UKY4), Caspase-3 (NP_001027871t), Caspase-6 (Augustus g16014.t2), Caspase-7 (Uniprot H2U497 and H2U498), Caspase-8 (Uniprot H2TIG5), Caspase-9 (Uniprot H2V836), Caspase-10 (Uniprot H2V630 and H2V635), Caspase-17 (Uniprot H2VEU2), Caspase-20 (Uniprot H2THL4), *Gasterosteus aculeatus* Caspase-1 (Uniprot G3P6H8), Caspase-1l (Augustus g16792.t3), Caspase-2 (Uniprot G3P808), Caspase-3a (Augustus g8732.t3), Caspase-3b (Uniprot G3Q4K5), Caspase-3c (Uniprot G3Q4L2 and BT027477), Caspase-6 (Uniprot G3PZL5), Caspase-7 (Augustus g18074.t1), Caspase-8 (NP_001254591), Caspase-9 (Augustus g27457.t1), Caspase-10 (NP_001254593), Caspase-17 (Augustus g16285.t1) and Caspase-20 (Augustus g4015.t1). Multiple alignment of 90 caspase protein sequences was generated with Muscle [52] and visualized with Jalview software [53]. The evolutionary history was inferred by using the Maximum Likelihood method based on the Whelan And Goldman + Freq. model [54]. Initial trees for the heuristic search were obtained by applying the Neighbor-Joining method to a matrix of pairwise distances estimated using a JTT model. A discrete Gamma distribution was used to model evolutionary rate differences among sites (5 categories (+G, parameter = 1.4388)). 500 bootstrap replications were used as a test of phylogeny, with values indicated next to the branch. The tree is drawn to scale, with branch lengths measured in the number of substitutions per site.

### RT-PCR

cDNA was prepared from different developmental stages as mentioned above and used for RT-PCR. Full length primers (Table 1) were used to amplify *casp1*, *casp3a*, *casp3b*, *casp6a*, *casp6b*, *casp7*, *casp17*, *casp19a*, *casp19b*, *casp21* and *casp22*. Different primers were used for the following caspases: *casp2*_fw: TAATGTGAGGTTCGACTCAG; *casp6c*_rv: TCCAGTTGTGAACGATAACG; *casp8a*_fw: CTACGATGTGATAATTCTCGT; *casp8b*_fw: ACAGATGAACCGAAGAGG; *casp9*_fw: AATACAGAGCAAGGCAACC; *casp10*_fw: CACGAGACCTCAACACTG; *casp10*_rv: GTGAATGTCTGAGGAAACAC; *casp20*_fw: TGACTACAATAACCTCTCTGATG; and *casp23*_fw: TCCAATTCTGAAGTGCAACT. The following primers were used to amplify *actin* as a control: *actin_fw*: ATCCCAAAGCCAACAGAGAG; *actin_rv*: CAACGGAAACGCTCATTGC. PCR reactions were conducted using 30 to 36 cycles.

### In situ hybridization

*In vitro* transcription of digoxigenin-labeled probes was performed using the RNA Labeling Kit (Roche Diagnostics Corporation) according to manufacturer’s instructions. Embryos were dechorionated at the appropriate developmental stages and fixed in 4% paraformaldehyde in phosphate buffered saline (pH 7.4) for 2 hours at room temperature and overnight at 4°C. Whole-mount in situ hybridization was performed as previously described [55]. Sense probes were used as controls for all caspases and did not reveal any staining. After staining, embryos were cleared in 80% glycerol for imaging. Images were acquired using an Olympus SZX16 stereomicroscope equipped with an Olympus DP80 dual color camera and Cellsens standard software. Digital images were cropped and aligned using Adobe Photoshop.

## Results and discussion

### Identification of zebrafish caspases

13 caspase genes (*casp*) have been identified in human, including *casp*1 to 10, 12, 14 and 16p (*casp16p* being a pseudogene) [16, 17]. Additional caspases have been detected in other vertebrates and include mammalian *casp15* (absent in the human and mouse genomes) [15], *casp17* (present in all vertebrate lineages except for therian mammals), and *casp18* (present in chicken but absent in placental mammals) [16]. We blasted these sequences against GenBank and Ensembl *Danio rerio* (GRCz10) EST databases to identify corresponding zebrafish caspases and found 19 *casp* genes (Table 2). Some of these genes were reported previously but were not always named according to the accepted caspase nomenclature [16, 56–59]. Other genes we identified have never been described before. We named all caspase genes according to their similarity to the vertebrate orthologs, and attributed new numbers based on the taxonomy proposed in previous studies [16]. The accession numbers of all genes from the different databases are shown in Table 2, and new GenBank accession numbers were obtained for all sequences.

**Table 2:** Nomenclature and accession numbers of zebrafish caspases.

Gene name	other / previous names	GRCz10 chromosome location (strand)	Ensembl Gene ID	Genbank Acc # (old)	Genbank Acc # (new)
*casp1*	*caspase-a*, *caspy*, *zgc*:*109869*	chr16:42,043,825–42,054,428 (-)	ENSDARG00000008165	BC095022	MG957992
casp19a*	*caspase-b*, *caspy2*,* zgc*:*109807* *caspase-1A*, *si*:*ch211-233g6*.*8*	chr1:57,315,773–57,323,452 (-)	ENSDARG00000052039 ENSDARG00000075270	BC095000, DQ022755	MG958005
casp19b*	*caspase-bl*, *caspase-b1*, *si*:*ch211-15j1*.*6*	chr1:57,370,656–57,376,144 (-)	ENSDARG00000094433	-	MG958006
casp23*	*caspase-c*, *zgc*:*113326*, *zgc*:*171731*	chr7:19,348,435–19,352,383 (+)	ENSDARG00000014657	BC151948	MG958010
*casp2*	*caspase-2*	chr16:17,643,307–17,669,604 (-)	ENSDARG00000062052	BC163115	MG957993
*casp9*	*im*:*7136887*,* zgc*:*101776*	chr23:25,047,448–25,058,524 (+)	ENSDARG00000004325	BC097103	MG958002
*casp8a*	*zgc*:*92075*	chr6:12,811,868–12,821,422 (+)	ENSDARG00000058325	BC081583	MG958000
*casp8b*	*caspase-8l1*	chr6:12,862,945–12,867,126 (+)	ENSDARG00000058341	DQ812121	MG958001
*casp10*	*caspase-8l2*, *caspase-8b*	chr9:1,343,221–1,355,521 (+)	ENSDARG00000070272	DQ812123 (partial)	MG958003
casp20*	*caspxa*,*caspxb*, *zgc*:*194469*, *CARD-Casp8*	chr6:12,878,158–12,881,286 (-)	ENSDARG00000058347	BC163666	MG958007
casp22*	*si*:*dkey-103e21*.*5*	chr5:22,081,469–22,086,320 (-)	ENSDARG00000091926	BC133883	MG958009
*casp3a*	*caspase-3a*, *zgc*:*100890*	chr1:16,835,237–16,841,582 (+)	ENSDARG00000017905	BC078310	MG957994
*casp3b*	*caspase-3b*	chr14:4,022,953–4,036,126 (-)	ENSDARG00000055045	DQ812120	MG957995
*casp6a*	*caspase-6a*, *zgc*:*112960*	chr3:32,830,371–32,834,050 (-)	ENSDARG00000093405	BC094299	MG957996
*casp6b*	*caspase-6b*, *zgc*:*103604*	chr3:32,822,780–32,826,756 (-)	ENSDARG00000025608	BC083437	MG957997
*casp6c*	*caspase-6c*, *zgc*:*136946*	chr3:32,837,963–32,841,789 (-)	ENSDARG00000070368	BC114318	MG957998
*casp7*	* zgc*:*110595*	chr12:30,456,171–30,467,659 (-)	ENSDARG00000091836	BC095327	MG957999
casp21*	*CABZ01041682*.*1; FO834888*.*1*	chr21:8,440,994–8,445,761 (+)	ENSDARG00000055550	-	MG958008
*casp17*	*CU467905*.*1*	chr10:42,334,831–42,350,374 (+)	ENSDARG00000086266 (partial)	-	MG958004

* new number attributed

**Fig 1:**
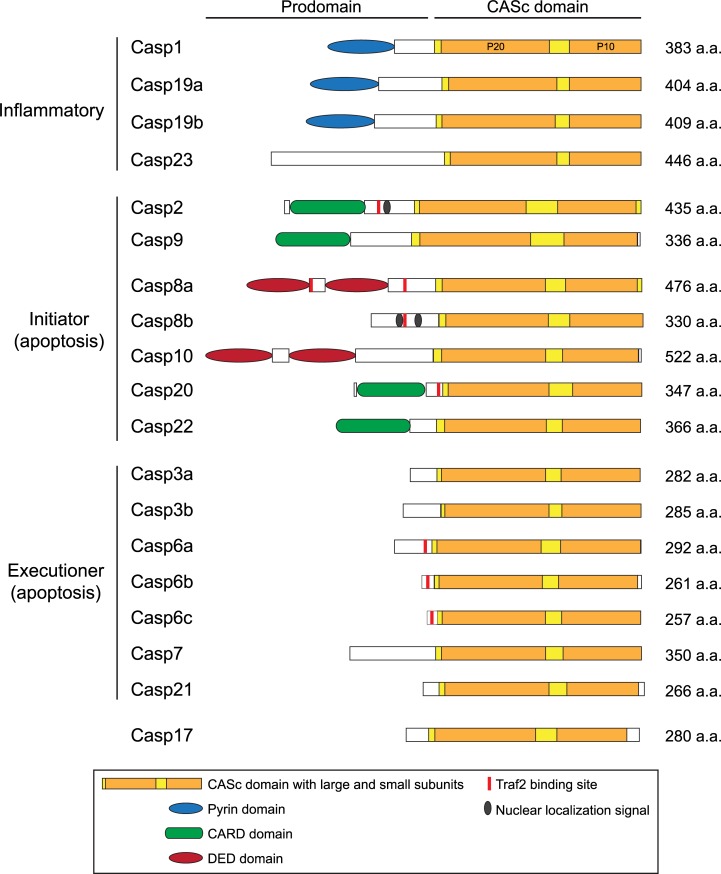
Domain structure of zebrafish caspases. Caspases are presented based on the classical classification of caspases as inflammatory, initiator, or executioner. The catalytic CASc domain is indicated in yellow, with large and small subunits in orange. CARD: caspase-recruitment domain; DED: death-effector domains.

**Fig 2:**
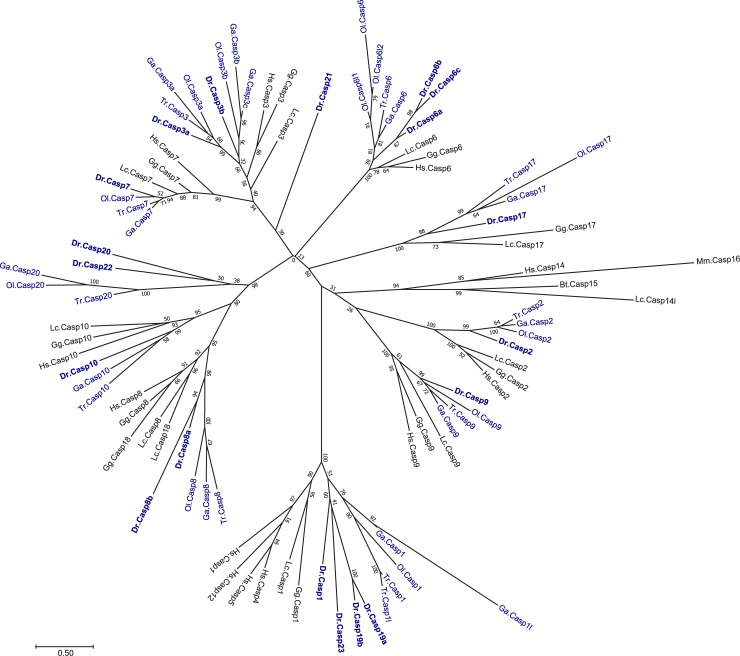
Phylogenetic tree of zebrafish and other relevant vertebrate caspases. A phylogenetic comparison was conducted for caspase protein sequences from zebrafish (Dr), human (Hs), mouse (Ms, used for Casp16), cow (Bt, used for Casp15), chicken (Gg), coelacanth (Lc), medaka (Ol), stickleback (Ga) and fugu (Tr). Teleost species are indicated in blue, with zebrafish in bold. 500 bootstrap replications were used as a test of phylogeny, with values indicated next to the branch.

**Fig 3:**
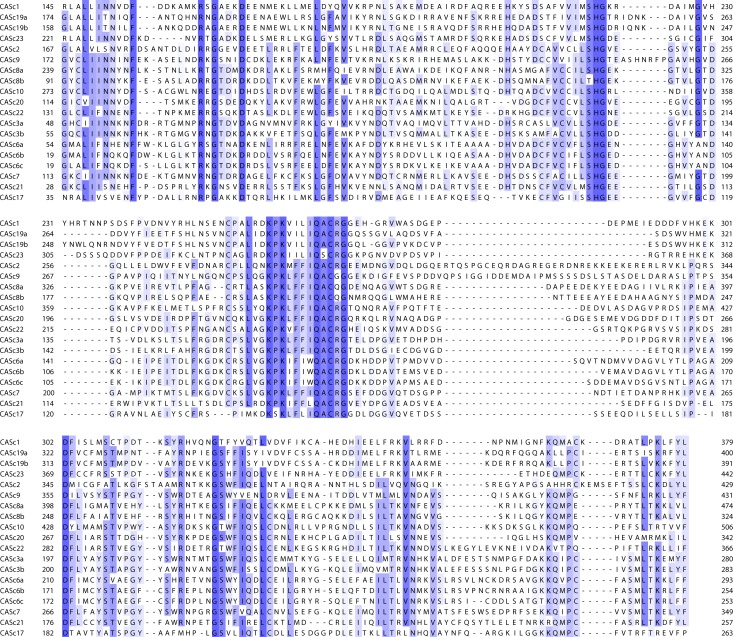
Multiple protein sequence alignment of zebrafish caspase CASc catalytic domains. Residues highlighted in blue are conserved across caspases.

For simplicity, we analyzed zebrafish caspases based on the initial classification of caspases into inflammatory, initiator and executioner groups (Fig 1). In human, genes encoding inflammatory caspases include *casp1*, *casp4*, *casp5*, and *casp12* and are clustered on chromosome 11, suggesting they may have originated from gene duplication events. In contrast, we found zebrafish *casp1*, *casp19a*, *casp19b* and *casp23* genes on three different chromosomes (Table 2) and did not observe any conserved chromosomal synteny with human caspases (data not shown). Surprisingly, we could only find one or two inflammatory caspase genes in other teleost species (*casp1* in medaka and *casp1/1l* in stickleback and fugu) and did not identify any *casp23* ortholog. Phylogenetic analysis of caspase sequences across vertebrates revealed that zebrafish inflammatory caspases cluster in a separate group, suggesting the occurrence of duplication events specific to that lineage (Fig 2). Interestingly, comparison of caspase protein sequences shows that zebrafish Caspase-1 (previously known as Caspy [56]) is the most similar to human Caspase-1, sharing 38% identity and 56% homology (Table 3). Analysis of the conserved catalytic CASc domain also indicates that zebrafish Caspase-1 has a His at position 318 that is not conserved in Caspases-19a, -19b and -23 (Fig 3). His 318 is similar to His 342 in human Caspase-1 that binds the specific P3 alanine residue of Caspase-1 substrates [60], suggesting functional conservation. Caspases-19a and 19b are 70% identical (Table 4) and phylogenetically cluster together, indicating they are recent duplicated isoforms. While mammalian inflammatory caspases are characterized by the presence of a caspase-recruitment domain (CARD) in their N-terminal region [61], Caspases-1, -19a and -19b share a pyrin (PYR) domain in its place. Interestingly, these PYR domains appear specific to zebrafish caspases, as Caspases-1 and -1l in other teleosts possess a CARD domain similar to mammals. Although in the same group, Caspase-23 differs from the other inflammatory caspases by the absence of a PYR or CARD domain in its N-terminal region (Fig 1). Caspase-23 also possesses an unusual QSCRG cysteine active site (positions 340–344) in its CASc domain instead of the conserved QACRG pentapeptide found in other caspases (Fig 3). Interestingly, a similar QSCRG is found in amphoxius and sea snail caspases [62, 63], suggesting an ancient origin.

**Table 3:** Protein sequence identity (%) and similarity (%) with other vertebrate caspases.

	Hs. CASP1	Hs. CASP2	Hs. CASP3	Hs. CASP4	Hs. CASP5	Hs. CASP6	Hs. CASP7	Hs. CASP8	Hs. CASP9	Hs. CASP10	Hs. CASP12	Hs. CASP14	Bt. CASP15	Mm. CASP16	Gg. CASP17	Gg. CASP18
**Casp1**	**38/55**	29/47	27/45	35/56	33/51	24/42	25/48	26/42	30/49	23/40	32/51	28/42	26/47	23/47	19/33	26/44
**Casp19a**	33/56	25/49	25/41	32/54	30/53	19/41	26/51	24/42	27/49	24/45	30/50	23/39	26/46	25/48	20/36	24/46
**Casp19b**	32/52	26/49	27/42	33/55	31/49	21/39	26/49	24/42	27/46	23/42	33/50	25/41	27/48	25/50	19/34	27/47
**Casp23**	33/50	26/46	23/38	31/50	31/51	20/35	22/41	21/42	23/41	22/44	28/44	20/34	24/42	22/40	18/33	24/48
**Casp2**	26/47	**48/65**	27/40	26/47	28/51	25/40	26/45	27/44	32/52	27/45	26/42	24/36	27/43	26/45	22/32	24/48
**Casp9**	25/47	30/52	29/42	24/45	25/45	27/41	31/49	30/47	**49/67**	30/49	22/42	23/38	27/45	26/44	20/35	30/52
**Casp8a**	27/46	28/49	27/39	27/45	27/47	26/38	29/45	**38/55**	32/50	37/56	23/41	22/34	25/42	25/41	21/34	40/60
**Casp8b**	28/46	25/42	31/53	27/48	25/41	31/49	30/48	**30/43**	27/46	29/43	25/46	26/43	25/45	25/39	22/39	32/46
**Casp10**	24/43	25/46	25/37	23/41	24/47	22/36	30/44	35/57	30/46	**37/60**	22/39	22/33	23/41	24/42	19/33	33/52
**Casp20**	28/47	30/44	34/48	28/46	27/43	28/44	30/51	29/43	31/51	32/46	26/47	28/42	28/47	23/44	21/38	29/46
**Casp22**	31/50	27/48	29/46	28/50	27/46	29/46	32/51	32/46	34/52	33/48	30/51	29/43	27/47	25/49	27/42	31/52
**Casp3a**	27/40	25/40	**56/71**	29/42	24/37	38/58	41/54	24/36	28/45	23/36	28/42	33/47	27/45	23/41	29/48	26/38
**Casp3b**	27/42	23/40	**56/70**	29/42	25/39	38/54	45/57	26/36	27/44	24/36	27/46	31/49	26/43	20/38	28/50	26/37
**Casp6a**	26/38	24/38	41/61	28/42	24/40	**65/77**	33/48	25/37	29/44	23/34	26/45	29/50	27/45	24/42	27/42	25/38
**Casp6b**	19/36	23/34	37/60	25/40	20/35	**53/68**	29/43	20/32	26/42	21/33	21/42	30/54	25/42	22/40	29/51	22/34
**Casp6c**	22/36	23/35	39/59	25/40	22/34	**54/67**	29/41	25/32	26/42	22/34	22/43	32/54	25/41	23/40	28/50	25/36
**Casp7**	28/45	28/45	46/60	29/48	29/45	37/51	**54/67**	30/45	30/50	28/42	27/47	29/45	26/47	23/43	27/45	31/44
**Casp21**	25/41	23/38	38/60	29/42	25/37	37/51	32/46	20/33	24/40	24/35	28/44	31/47	26/41	22/37	32/53	26/39
**Casp17**	20/37	21/35	28/49	21/38	19/36	27/49	28/44	21/34	21/36	21/35	21/39	28/44	22/40	22/35	**37/54**	21/37

Highest percentages of identity and similarity are highlighted in grey.

Identity and similarity between orthologs are indicated in bold.

Hs: *Homo sapiens*; Mm: *Mus musculus*; Bt: *Bos Taurus*; Gg: *Gallus gallus*

**Table 4:** Protein sequence identity (%) and similarity (%) among zebrafish caspases.

	**Casp1**																		
**Casp1**	**100**	**Casp19a**																	
**Casp19a**	41/61	**100**	**Casp19b**																
**Casp19b**	42/60	**70/82**	**100**	**Casp23**															
**Casp23**	36/52	33/52	34/53	**100**	**Casp2**														
**Casp2**	26/48	26/50	27/45	26/46	**100**	**Casp9**													
**Casp9**	28/48	27/45	27/48	24/46	30/54	**100**	**Casp8a**												
**Casp8a**	26/45	25/46	25/45	23/46	31/52	29/49	**100**	**Casp8b**											
**Casp8b**	25/50	25/47	23/44	22/39	29/44	30/46	40/53	**100**	**Casp10**										
**Casp10**	23/40	28/45	26/44	25/46	29/46	30/49	35/53	28/39	**100**	**Casp20**									
**Casp20**	29/47	24/46	23/45	25/43	28/46	31/50	34/47	35/56	31/43	**100**	**Casp22**								
**Casp22**	29/51	24/51	26/51	26/45	30/49	31/52	33/50	33/54	33/47	38/55	**100**	**Casp3a**							
**Casp3a**	26/42	23/39	22/40	24/35	26/41	27/42	28/40	31/51	25/38	32/48	32/47	**100**	**Casp3b**						
**Casp3b**	26/45	25/42	26/40	23/34	26/40	26/41	25/39	32/52	23/37	32/49	33/49	**61/77**	**100**	**Casp6a**					
**Casp6a**	25/43	20/40	24/41	23/37	26/40	27/42	26/38	31/48	24/36	31/49	30/45	38/57	39/59	**100**	**Casp6b**				
**Casp6b**	22/38	21/39	18/38	21/34	24/36	26/35	25/36	30/46	20/35	31/46	28/43	37/57	36/54	**66/77**	**100**	**Casp6c**			
**Casp6c**	25/40	20/39	20/38	22/31	24/36	27/40	26/35	32/46	23/35	31/45	31/45	36/55	36/54	**63/73**	**80/89**	**100**	**Casp7**		
**Casp7**	25/48	27/49	26/47	25/42	27/44	32/47	30/46	33/56	30/44	35/54	33/52	47/62	49/63	37/53	35/48	35/47	**100**	**Casp21**	
**Casp21**	27/42	26/41	24/39	25/37	24/37	25/39	21/36	33/51	22/36	30/45	28/44	42/60	40/60	34/52	37/56	37/56	37/52	**100**	**Casp17**
**Casp17**	20/37	20/37	20/37	19/35	23/38	21/34	22/35	28/48	20/34	28/42	26/43	31/52	29/53	28/50	31/47	31/45	27/49	34/53	**100**

Identity and similarity between duplicated isoforms are highlighted in grey and bold.

Genes encoding initiator caspases in mammals include *casp2*, *casp9*, and the subfamily of *casp8* and *casp10*. Another caspase belonging to the caspase-8 subfamily, *casp18*, has been detected in chicken and opossum but is not present in eutherian mammals [16]. As previously reported [17, 58, 59, 64], we identified orthologs for *casp2*, *casp9*, *casp8* (as *casp8a* and *casp8b*), and *casp10* but not for *casp18* in zebrafish (Tables 2 and 3, Fig 2). We also detected additional caspase genes belonging to the caspase-8 subfamily that we named *casp20* and *casp22*. Like in mammals, zebrafish Caspase-2 and Caspase-9 are characterized by the presence of a CARD in their N-terminal region (Fig 1). Caspase-8a and Caspase-10, on the other hand, possess two death-effector domains (DEDs). Interestingly, the N-terminal pro-domain of Caspase-8b is much shorter and lacks these DED motifs. It notably includes two nuclear localization signals not detected in Caspase-8a (Fig 1). Despite these differences, the CASc domains of Caspase-8a and Caspase-8b are very similar (62% identity and 77% homology) and possess the QACQG active site characteristic of human Caspase-8 (Fig 3), indicating that both caspases are duplicated isoforms. The adjacent localization of *casp8a* and *casp8b* genes on chromosome 6 further suggests a common origin from a recent gene duplication event. This duplication appears specific to the zebrafish lineage, as no *casp8b* could be identified in other teleosts including fugu, medaka and stickleback (S1 Fig). In addition to *casp8a* and *casp8b*, we detected another paralog on chromosome 6 that we named *casp20*. This paralog has been described in previous studies as CARD-Casp8 due to the presence of a CARD in the N-terminal pro-domain instead of the two DED domains present in Caspases-8, -10 and -18 [58, 59] (Fig 1). Interestingly, comparative genomics and phylogenetic analyses suggest that *casp8*, *casp10*, *casp18* originate from a common ancestor during vertebrate evolution (Fig 2) [59]. *Casp8*, *casp18* and *casp10* genes are clustered on the same chromosome in chicken, coelacanth and spotted gar genomes (S1 Fig and [59]). While *casp18* has been lost in the human genome (and in other eutherian mammals), *casp8* and *casp10* remain clustered on chromosome 2 (S1 Fig). In contrast, *casp8* and *casp10* have been segregated on different chromosomes in the teleost lineage after extensive chromosomal rearrangements during evolution (S1 Fig). Similarly to *casp18* in other species, *casp20* is found in close proximity to *casp8* in all teleost genomes (*casp8b* in zebrafish) but could not be identified in other vertebrates including shark, lamprey and gar. The clustering of *casp8*, *casp18* and *casp10* in the genome of spotted gar, whose lineage represents the unduplicated sister taxon of teleosts [65], suggests that *casp8*, *casp18* and *casp10* genes formed an ancestral cluster in vertebrates that has been dispersed during teleost evolution. *Casp20* might have derived from *casp18* by domain shuffling from DED to CARD or might have arisen as a new gene after duplication in teleosts. In addition to *casp20*, we discovered a previously unknown caspase on chromosome 5 that we named *casp22* (Fig 2, Table 2). Surprisingly, we could not identified any *casp22* ortholog in other teleost genomes. Analysis of the chromosomal synteny around the *casp22* locus revealed a partial conservation among teleosts, with *casp22* neighboring genes being dispersed on different chromosomes in species other than zebrafish (data not shown). Like Caspase-20, Caspase-22 possesses a CARD in its N-terminal region (Fig 1). Phylogenetic analysis revealed that *casp20* and *casp22* segregate together (Fig 2), suggesting they might originate from a duplication event unique to the zebrafish lineage.

The last classical functional group of caspases in vertebrates includes apoptosis executioners Caspases-3, -6 and -7 that are characterized by a short pro-domain. Another caspase sharing a similar structure, Caspase-17, has been identified in vertebrates other than therian mammals [16], but its function in programmed cell death has not been defined. We identified two orthologs for *caspase-3* (*casp3a* and *casp3b*), three orthologs for *caspase-6* (*casp6a*, *casp6b* and *casp6c*), and one ortholog each for *caspase*-7 (*casp7*) and *caspase-17* (*casp17*) (Fig 2 and Table 3). We also discovered a novel uncharacterized caspase on chromosome 21 that we named *casp21* (Table 2). Protein sequence comparison revealed a high level of conservation between human and zebrafish Caspase-3, -6 and -7 (Table 3). *Casp3a* and *casp3b* are located on different chromosomes, suggesting they have arisen from the whole genome duplication that occurred in the teleost lineage [66]. Supporting that hypothesis, several *casp3* genes were also found on different chromosomes in medaka and stickleback and clustered in two distinct groups in our phylogenetic analysis (Fig 2). We could only identify one *casp3* in fugu and tetraodon, suggesting a specific gene loss in the pufferfish lineage. In contrast to *casp3a* and *casp3b*, *casp6a*, *casp6b* and *casp6c* were found in adjacent positions on chromosome 3. Interestingly, we only identified one *casp6* gene in stickleback, fugu or tetraodon, but three potential *casp6* paralogs on independent scaffolds in medaka. Phylogenetic analysis showed that zebrafish and medaka *casp6* paralogs cluster in separate groups, suggesting independent gene duplication events in these two lineages. Analysis of the chromosomal synteny at the casp6 locus further revealed a conservation of *casp6* flanking genes among teleosts except in zebrafish, suggesting extensive chromosomal rearrangements in addition to duplications (S2 Fig). Interestingly, the three zebrafish Caspase-6s share a Traf2 (TNF receptor associated factor 2) binding site in their pro-domain that is also found in Caspases-8a, -8b and -2 but not in other executioner caspases (Fig 1), suggesting they might participate in the regulation of tumor necrosis factor (TNF) signaling. As reported previously [16], we also identified *casp17* as a distinct caspase that does not phylogenetically segregate with the group of executioner caspases (Fig 2). Remarkably, Caspase-17 has a Met in its CASc domain at position 197 instead of the Arg conserved in all other caspases that is involved in substrate binding (Fig 3). Finally, we discovered a novel caspase, Caspase-21, that segregates with the group defined by Caspases-3 and -7 in our phylogenetic analysis (Fig 2). *Casp21* appears specific to the zebrafish lineage, as we could not identify any *casp21* ortholog in other teleost genomes. Protein sequence comparison between Caspase-21 and the other zebrafish caspases further showed a higher similarity with Caspase-3a and Caspase-3b (Table 4), suggesting they might share common functional properties.

### Spatiotemporal expression of caspases during development

To characterize the expression of caspases during development, we analyzed the temporal and spatial expression of all caspase genes from cleavage to larval stages using reverse transcription PCR (RT-PCR) and in situ hybridization (ISH).

#### Inflammatory caspases

**Fig 4:**
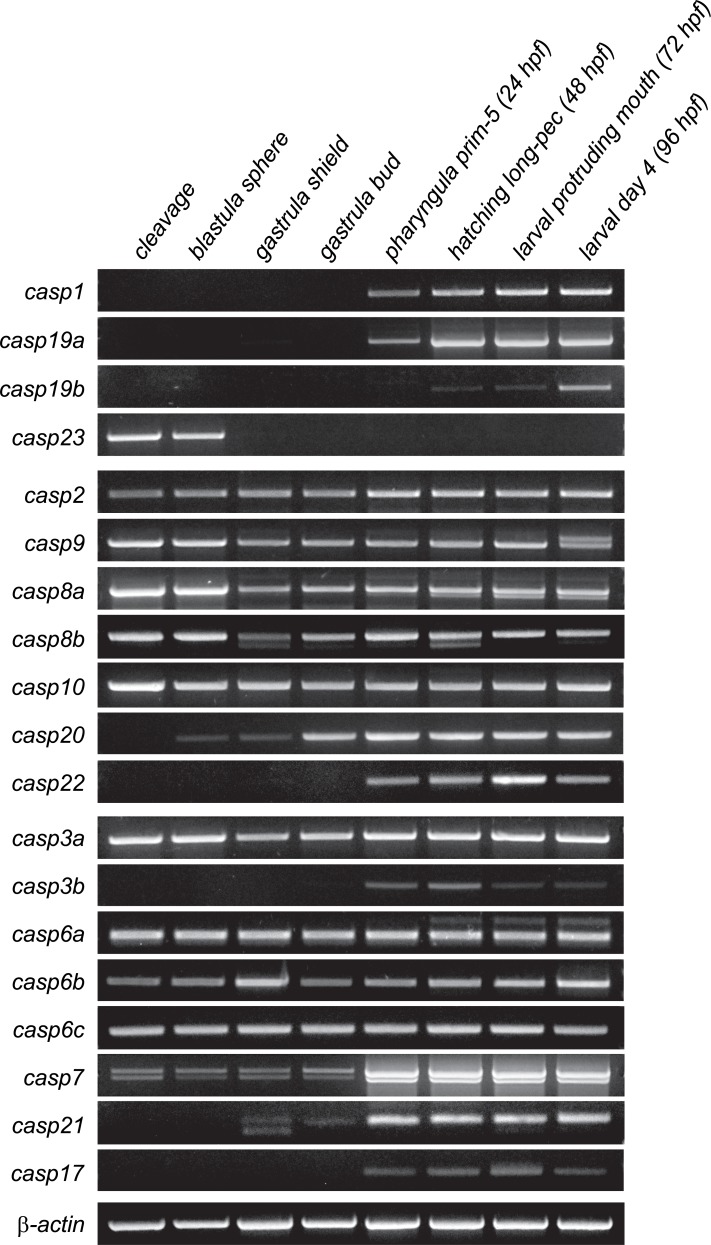
Temporal mRNA expression of caspases during embryonic development. RT-PCR was performed for all 19 caspase genes using cDNA from specified developmental stages. β-actin was used as a control.

**Fig 5:**
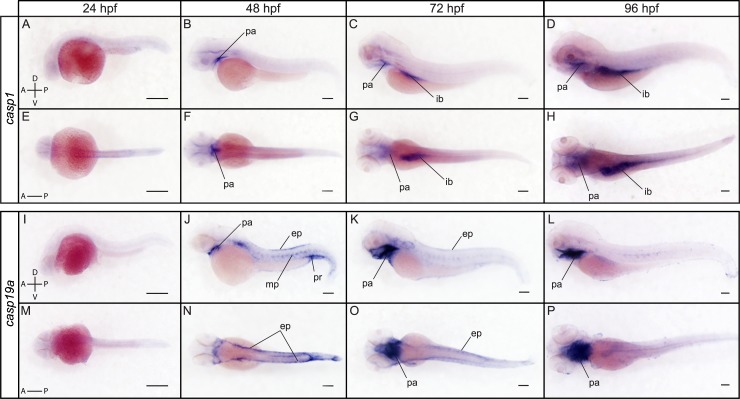
Spatial expression of *casp1* and *casp19a* at 24, 48, 72 and 96 hpf. Lateral (A-D) and dorsal (E-H) views of whole embryos stained for *casp1* by ISH show expression in the pharyngeal arches (pa) at 48, 72 and 96 hpf and in the intestinal bulb (ib) at 72 and 96 hpf. *Casp19a* expression is strongly detected in the pharyngeal arches (pa) at 48, 72 and 96 hpf, and is also seen in the epidermis (ep) at 48 and 72 hpf (lateral views in J and K, dorsal views in N and O). Expression is also observed in the proctodeum (pr) and at lower levels in the muscles pioneers (mp) at 48 hpf. Scale bar: 200 μm.

RT-PCR analysis revealed that *casp1*, *casp19a*, *casp19b* and *casp23* have different temporal expression profiles during development (Fig 4). Expression of *casp1* and *casp19a* begins at the pharyngula stage (24 hpf) and is maintained at 48, 72 and 96 hpf. In contrast, *casp19b* expression was only weakly detected at 48 and 72 hpf and became clearly visible at 96 hpf. Remarkably, *casp23* expression could only be detected at cleavage and sphere stages, indicating that *casp23* is only maternally expressed. ISH further revealed common and specific expression domains for *casp1* and *casp19a* at 48, 72 and 96 hpf (Fig 5). While both caspases were detected in the pharyngeal arches as previously described [56], *casp1*, but not *casp19a*, was also found in the intestinal bulb at 72 and 96 hpf (Fig 5C, 5D, 5G and 5H). In contrast, *casp19a* was specifically detected in the epidermis at 48 and 72 hpf as well as in the proctodeum at 48 hpf (Fig 5J–5O), suggesting distinct functions in these specific organs. Casp19a expression became notably restricted to the pharyngeal arches at 96 hpf (Fig 5L and 5P). Our attempts to detect *casp19b* with probes directed against the coding sequence or the 5’UTR of the transcript were unfortunately unsuccessful, suggesting that *casp19b* expression levels might be below the detection threshold of ISH.

#### Initiator caspases

Initiator caspases including *casp2*, *casp9*, and the members of the caspase-8 subfamily *casp8a*, *casp8b*, *casp10*, *casp20* and *casp22* share a similar temporal expression profile during development, as shown by RT-PCR (Fig 4). Expression of *casp2*, *casp9*, *casp8a*, *casp8b* and *casp10* was detected throughout embryonic development from cleavage to larval stages, indicating both maternal and zygotic expression. *Casp8a* and *casp8b* expression appeared notably stronger at maternal stages, suggesting an important function for these caspases during early development. *Casp20* expression was not detected at cleavage stage but was observed at very low levels at sphere and shield stages. It was then strongly detected from bud stage to 96 hpf. In contrast, *Casp22* expression was only observed at lower levels from 24 to 96 hpf.

**Fig 6:**
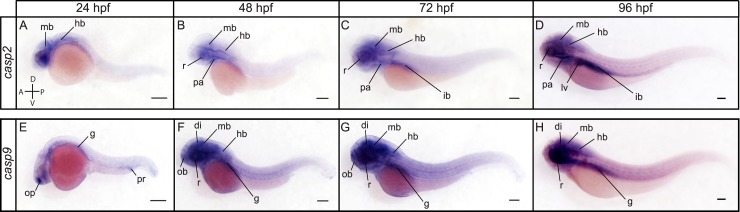
Spatial expression of *casp2* and *casp9* at 24, 48, 72 and 96 hpf. Lateral views of whole embryos stained for *casp2* (A-D) and *casp9* (E-H) by ISH. *Casp2* is expressed in the midbrain (mb) and hindbrain (hb) at all stages analyzed. Expression is also observed in the pharyngeal arches (pa) and retina (r) at 48, 72 and 96 hpf (B-D), and in the intestinal bulb (ib) at 72 and 96 hpf (C, D). *Casp2* becomes strongly detected in the liver (lv) at 96 hpf (D). *Casp9* is expressed at high levels in the olfactory placodes (op) and at lower levels in the gut (g) and proctodeum (pr) at 24 hpf (E). *Casp9* appears ubiquitously expressed at low levels at 48 and 72 hpf and is strongly detected in the retina, diencephalon (di), midbrain, hindbrain and gut from 48 to 96 hpf (F-H). Scale bar: 200 μm.

ISH revealed distinct expression patterns for *casp2* and *casp9* from 24 to 96 hpf (Fig 6). While *casp2* appeared selectively expressed in the midbrain and hindbrain at 24 hpf (Fig 6A), *casp9* expression was strongly detected in the olfactory placode and was observed at lower levels in the gut and proctodeum (Fig 6D). *Casp2* continued to be expressed in the midbrain and hindbrain at 48 and 72 hpf and was also detected in the retina and pharyngeal arches at these stages (Fig 6B and 6C). Expression in the intestinal bulb appeared at 72 hpf (Fig 6C). Compared to *casp2*, *casp9* appeared ubiquitously expressed at low levels at 48 and 72 hpf (Fig 6E and 6F). Stronger expression was detected in the retina and different regions of the brain including the diencephalon, midbrain and hindbrain, which is consistent with the reported role of Caspase-9 in retinal axon arbor dynamics [38]. At 96 hpf, *casp2* and *casp9* became more similarly expressed and were strongly detected in the intestinal bulb, the nervous system and the retina (Fig 6D and 6H). C*asp2*, but not *casp9*, was notably observed in the liver at that stage (Fig 6D).

**Fig 7:**
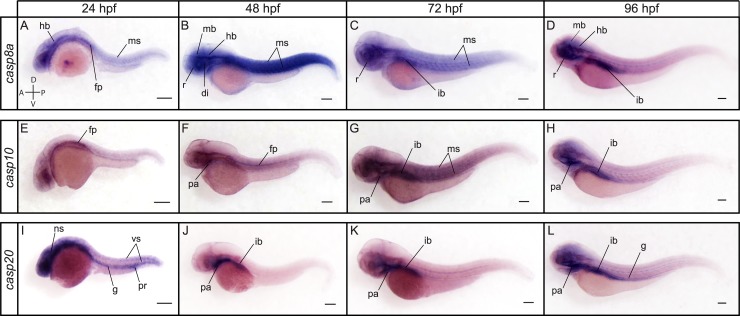
Spatial expression of the caspase-8 family members *casp8a*, *casp10* and *casp20* at 24, 48, 72 and 96 hpf. Lateral views of whole embryos stained for *casp8a* (A-D), *casp10* (E-H) and *casp20* (I-L) by ISH. *Casp8a* is expressed in the hindbrain (hb), muscles (ms) and at the floorplate (fp) at 24 hpf (A). Its expression is then strongly detected in the retina (r), diencephalon (di), midbrain (mb), hindbrain and muscles (ms) at 48 hpf (B). Additional expression in the intestinal bulb (ib) is observed at 72 and 96 hpf (C, D). *Casp10* expression is detected at the floorplate at 24 and 48 hpf (E, F) and in the pharyngeal arches (pa) at 48 hpf (F). A strong expression is detected in the pharyngeal arches, muscles and intestinal bulb at 72 hpf (G). *Casp10* remains highly detected in the pharyngeal arches and intestinal bulb at 96 hpf (H). *Casp20* is strongly expressed in the nervous system (ns), vascular system (vs), proctodeum (pr) and throughout the gut (g) at 24 hpf (I). Its expression becomes restricted to the pharyngeal arches and the intestinal bulb at 48, 72 and 96 hpf (J-L). Scale bar: 200 μm.

Members of the caspase-8 subfamily demonstrated a different spatial expression compared to *casp2* and *casp9* (Fig 7). While we were unable to detect *casp8b* and *casp22* despite using multiple probes directed against the coding sequence, 3’UTR, or 5’UTR of both transcripts, we observed a strong expression of *casp8a* in the muscles, retina and nervous system at 24, 48 and 72 hpf (Fig 7A–7C). *Casp8a* expression decreased in the muscles but remained high in the nervous system and retina at 96 hpf and became prominent in the intestinal bulb (Fig 7D). *Casp10* appeared to be expressed at lower levels and was notably detected at the floorplate at 24 and 48 hpf (Fig 7E and 7F). Its expression became apparent in the pharyngeal arches at 48 and 72 hpf (Fig 7F and 7G) and was strongly detected in the muscles and intestinal bulb at 72 hpf (Fig 7G). Similarly to *casp8a*, *casp10* expression decreased in the muscles and became strongly detected in the intestinal bulb at 96 hpf (Fig 7H). In contrast to the low expression of *casp10* at 24 hpf, *casp20* was strongly detected in the nervous system, throughout the gut and in the proctodeum at that stage (Fig 7I). Interestingly, *casp20* expression was also observed in the vascular system at this time point. At 48 hpf, however, *casp20* expression became restricted to the pharyngeal arches and the intestinal bulb (Fig 7J), where it remained strongly detected at 72 and 96 hpf (Fig 7K and 7L).

#### Executioner caspases

Known as the principal mediators of apoptosis in all tissues, executioner caspases appeared to have variable spatiotemporal patterns of expression during development. *Casp3a*, *casp6a*, *casp6b* and *casp6c* were detected at relatively constant levels from maternal to late stages of development by RT-PCR (Fig 4). In contrast, only low levels of expression were observed for *casp3b* and *casp17* from 24 to 96 hpf. *Casp7* expression was detected at low levels from cleavage to bud stages but increased from 24 to 96 hpf. Interestingly, a shorter *casp7* transcript was detected at all stages by RT-PCR and appeared to encode a protein with a shorter pro-domain. The functional significance of this isoform remains however unclear. *Casp21* was barely detected at shield and bud stages but became clearly expressed from 24 to 96 hpf.

**Fig 8:**
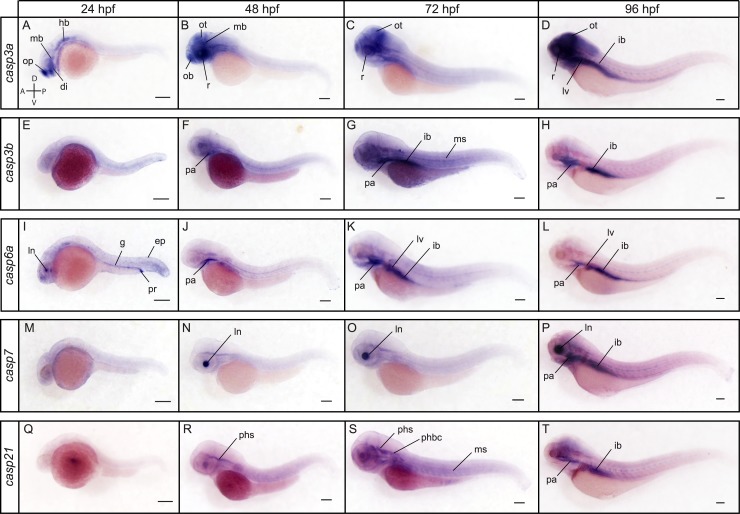
Spatial expression of the executioner caspases *casp3a*, *casp3b*, *casp6a*, *casp7* and *casp21* at 24, 48, 72 and 96 hpf. Lateral views of whole embryos stained for *casp3a* (A-D), *casp3b* (E-H), *casp6a* (I-L), *casp7* (M-P), and *casp21* (Q-T) by ISH. *Casp3a* is expressed in the olfactory placodes (op), diencephalon (di), midbrain (mb) and hindbrain (hb) at 24 hpf (A). Expression remains high in the nervous system at 48, 72 and 96 hpf and is strongly detected in the olfactory bulb (ob), retina (r), and optic tectum (ot) (B-D). *Casp3a* becomes strongly expressed in the intestinal bulb and liver at 96 hpf (D). *Casp3b* expression is not detected at 24 hpf (E) but becomes visible at 48 and 72 hpf, notably in the pharyngeal arches (pa), muscles (ms) and intestinal bulb (ib) (F, G). Expression becomes restricted to the pharyngeal arches and intestinal bulb at 96 hpf (H). *Casp6a* expression is mostly detected in the lens (ln), gut (g) proctodeum (pr) and epidermis (ep) at 24 hpf (I). It becomes restricted to the pharyngeal arches at 48 hpf (J), and is strongly detected in the pharyngeal arches, intestinal bulb and liver (lv) at 72 and 96 hpf (K, L). *Casp7* expression appears restricted to the lens at 48 and 72 hpf (N, O). It expands to the intestinal bulb at 96 hpf (P). *Casp21* expression is not visible at 24 hpf (Q) but is detected in the primary head sinus (phs) at 48 hpf (R). It is maintained in the primary head sinus and is also observed in the primordial hindbrain channel (phbc) and the muscles at 72 hpf (S). *Casp21* expression becomes visible in the nervous system, pharyngeal arches and intestinal bulb at 96 hpf (T). Scale bar: 200 μm.

**Fig 9:**
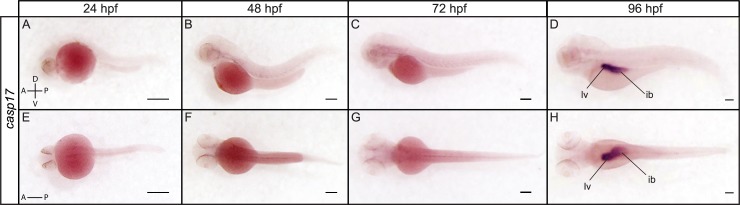
Spatial expression of *casp17* at 24, 48, 72 and 96 hpf. Lateral (A-D) and dorsal (E-H) views of a whole embryo stained for *casp17* by ISH show a strong and specific expression of *casp17* in the liver (lv) and intestinal bulb (ib) at 96 hpf (D, H). Scale bar: 200 μm.

ISH analysis revealed specific and complementary expression patterns of executioner caspases at 24, 48, 72 and 96 hpf (Fig 8). While *casp3b* could not be clearly detected at 24 hpf (Fig 8E), strong expression of *casp3a* was observed in the olfactory placodes, diencephalon, midbrain and hindbrain at that stage (Fig 8A). As reported in previous studies [38], *casp3a* remained strongly expressed in the brain and retina at 48, 72 and 96 hpf, but was not detected in the trunk (Fig 8B–8D). In contrast, *casp3b* appeared ubiquitously expressed, albeit at low levels, at 48 hpf, with a higher expression in the pharyngeal arches (Fig 8F). Expression levels appeared to increase at 72 hpf, being higher in the pharyngeal arches, muscles and intestinal bulb (Fig 8G). Both *casp3a* and *casp3b* became expressed at high levels in the intestinal bulb at 96 hpf (Fig 8D and 8H). While we have not been able to detect *casp6b* and *cas6c*, we observed a specific and dynamic expression of *casp6a* at 24, 48, 72 and 96 hpf. *Casp6a* expression was detected in the lens, gut, proctodeum and to a lower extent in the epidermis at 24 hpf (Fig 8I). It became restricted to the pharyngeal arches at 48 hpf (Fig 8J), but then expanded and was strongly visible in the liver and intestinal bulb at 72 and 96 hpf (Fig 8K and 8L). While *casp3a*, *casp3b* and *cas6a* are expressed in several tissues and organs, *casp7* was exclusively detected in the lens at 48 and 72 hpf (Figs 8N and 8O). Although surprising, such restricted expression has also been observed in the salmon embryo [67], suggesting a highly specific and conserved function among teleosts. Like *casp3a*, *casp3b* and *casp6a*, *casp7* expression became also detected in the intestinal bulb at 96 hpf (Fig 8P). *Casp21* expression was not detected at 24 hpf (Fig 8O) but became visible in the primary head sinus (phs) at 48 hpf (Fig 8R). Expression was maintained in the phs at 72 hpf and became also visible in the primordial hindbrain channel and the muscles (Fig 8S). It became detected in the pharyngeal arches and intestinal bulb at 96 hpf (Fig 8T). Finally, *casp17* expression could not be observed by ISH at 24, 48 or 72 hpf (Fig 9A, 9C, 9E and 9G) but was strongly and exclusively detected in the liver and intestinal bulb at 96 hpf (Fig 9D and 9H). Previous studies have also detected *casp17* in the liver of chicken [16], suggesting functional conservation among vertebrates.

## Conclusions

Our study reveals that the zebrafish caspase family is much larger than anticipated, including 19 distinct caspase genes. As summarized in Table 5, zebrafish caspases have both shared and specific expression profiles that suggest distinct and complementary functions during development. It is interesting to note that only a restricted number of caspases including *casp2*, *casp3a*, *casp8a*, *casp9* and *casp20* are strongly expressed in the developing nervous system. Other caspases may not be expressed there or may be expressed at such low levels in normal conditions that they would escape detection by ISH. We also discovered that some caspases such as *casp6a* or *casp19a* have dynamic expression patterns that change over time, while others such as *casp7* are unexpectedly expressed in a restricted number of structures or tissues. Altogether, our analysis offers a starting point for future studies investigating the functions of caspases during embryonic development. Our characterization of zebrafish caspases will be especially useful for the generation of new caspase mutants or the development of genetically encoded biosensors expressed in a cell- or tissue-specific manner.

**Table 5:** Developmental expression patterns of zebrafish caspases.

** **	** **	** **	ep	op/ob	di	mb	hb	fp	r	ln	mp/ms	phs/phbc	vs	pa	lv	ib	g	pr
INFLAMMATORY	*casp1*	24 hpf	** **	** **	** **	** **	** **	** **	** **	** **	** **	** **	** **	** **	** **	** **	** **	** **
		48 hpf												+++				
		72 hpf												+++		+++		
		96 hpf												+++		+++		
	*casp19a*	24 hpf																
		48 hpf	+++								+++			+++				+++
		72 hpf	+											+++				
		96 hpf												+++				
INITIATOR	*casp2*	24 hpf				++	++											
		48 hpf				++	++		++					++				
		72 hpf				+++	+++		+++					++		+++		
		96 hpf				+++	+++		+++					+++	+++	+++	+	
	*casp9*	24 hpf		+++													+++	+++
		48 hpf		+++	+++	+++	+++		+++		+						+++	
		72 hpf		+++	+++	+++	+++		+++		+						+++	
		96 hpf			++	++	++		+++					+		++	++	
	*casp8a*	24 hpf			+	+	+++	+++	+		+							
		48 hpf			+++	+++	+++		+++		+++							
		72 hpf				+	+		+++		+++					+++		
		96 hpf				++	++		+++					++		+++		
	*casp10*	24 hpf						++										
		48 hpf									+			+++				
		72 hpf									+++			+++		+++		
		96 hpf				++	++							+++		++		
	*casp20*	24 hpf		+++	+++	+++	+++						++				+++	+++
		48 hpf												+++		+++		
		72 hpf												+++		+++		
		96 hpf			++	++	++							+++		+++	+	
EXECUTIONER	*casp3a*	24 hpf		+++	+++	+++	+++											
		48 hpf		+++	+++	+++	++		+++									
		72 hpf		++	+++	+++	++		+++									
		96 hpf		++	+++	+++	++		+++						+++	++	++	
	*casp3b*	24 hpf																
		48 hpf									+			+				
		72 hpf									++			++		+++		
		96 hpf												++		+++		
	*casp6a*	24 hpf	+							++							+++	+++
		48 hpf												+++				
		72 hpf									+			+++	+++	+++		
		96 hpf												+	++	+++		
	*casp7*	24 hpf																
		48 hpf								+++								
		72 hpf								+++								
		96 hpf								+++				+++		+++		
	*casp21*	24 hpf																
		48 hpf									+	+						
		72 hpf									+	+++						
		96 hpf				+	+							++		++		
OTHER	*casp17*	24 hpf																
		48 hpf																
		72 hpf																
		96 hpf													+++	+++		

Expression levels detected by ISH are indicated by +, ++ or +++. ep: epidermis; op/ob: olfactory placodes / bulb; di: diencephalon; mb: midbrain; hb: hindbrain; fp: floorplate; r: retina; ln: lens; mp/ms: muscle pioneers/muscles; phs/phbc: primary head sinus/ primordial hindbrain channel; vs: vascular system; pa: pharyngeal arches; lv: liver; ib: intestinal bulb; g: gut; pr: proctodeum.

## Supporting information

S1 Fig Syntenic conservation between *casp8*, *casp10*, *casp18* and *casp20* orthologs. Caspase genes are represented in red. Genes conserved among coelacanth and tetrapods are represented in green while genes conserved among teleosts are shown in blue. Non-conserved genes are in white. Chromosomes are indicated on the left for each species, with zebrafish chromosomes highlighted in grey. Hs: *Homo sapiens*, Gg: *Gallus gallus*; Lc: *Latimeria chalumnae*, Dr: *Danio rerio*, Tr: *Takifugu rubripes*, Ga: *Gasterosteus aculeatus*, Ol: *Oryzias latipes*.
(EPS)

S2 Fig Syntenic conservation between *casp6* orthologs. Caspase-6 genes are represented in red. Genes conserved among coelacanth and tetrapods are represented in green while those conserved among teleosts are shown in blue. Non-conserved genes are in white. Chromosomes are indicated on the left for each species, with zebrafish chromosomes highlighted in grey. Hs: *Homo sapiens*, Gg: *Gallus gallus*; Lc: *Latimeria chalumnae*, Dr: *Danio rerio*, Tr: *Takifugu rubripes*, Ga: *Gasterosteus aculeatus*, Ol: *Oryzias latipes*, Tn: *Tetraodon nigroviridis*.
(EPS)
